# Clinical efficacy and safety of removing blood stasis and removing phlegm in the treatment of epilepsy with cognitive impairment: A protocol for systematic review and meta-analysis

**DOI:** 10.1097/MD.0000000000027929

**Published:** 2021-11-24

**Authors:** YangYang Yu, CanXing Yuan, Chao Gu

**Affiliations:** Department of Neurology, Longhua Hospital, Shanghai University of Traditional Chinese Medicine, Shanghai, China.

**Keywords:** effectiveness, epilepsy with cognitive impairment, removing blood stasis and phlegm, safety

## Abstract

**Background:**

Epilepsy is a chronic encephalopathy caused by abnormal discharge of neurons in the brain, resulting in brain dysfunction. Cognitive impairment is one of the most common complications of epilepsy. The current treatment of epilepsy in the control of symptoms at the same time cause a lot of side effects, especially the aggravation of cognitive impairment. Many literatures have stated that the efficacy and safety of integrated traditional Chinese and western medicine in the treatment of epilepsy with cognitive impairment is superior to that of western medicine alone. In this systematic review, we intend to evaluate the clinical efficacy and safety of removing stasis and resolving phlegm in the treatment of epilepsy with cognitive impairment.

**Methods:**

We will search The Cochrane Library, EMbase, Pubmed, Web of Science, Chinese Journal Full-Text Database (CNKI), Wanfang Database, and VIP database. Simultaneously we will retrieval relevant meeting minutes, eligible research reference lists, symposium abstracts, and gray literatures. We will not apply any restrictions to the language and publication date. All randomized controlled trials about the efficacy and safety of removing blood stasis and phlegm in the treatment of epilepsy with cognitive impairment will be included. Two authors will independently carry out. Any objections will be worked out by a third author through consultation. We will use the Revman 5.3 and Stata 13.0 software for data synthesis, sensitivity analysis, meta regression, subgroup analysis, and risk of bias assessment. The grading of recommendations assessment, development, and evaluation standard will be used to evaluate the quality of evidence.

**Results:**

This systematic review will synthesize the data from the present eligible high quality randomized controlled trials to assess whether the treatment of removing blood stasis and phlegm is effective and safety for epilepsy with cognitive impairment from various evaluation aspects including clinical efficacy of epilepsy, EEG improvement rate, MOCA score, QOLIE-31 cognitive function score, traditional Chinese medicine symptom score, incidence of adverse reactions, frequency of seizures of epilepsy, and duration of seizure of epilepsy.

**Conclusion:**

The systematic review will provide evidence to assess the efficacy and safety of removing blood stasis and phlegm in the treatment of patients with epilepsy with cognitive impairment.

**PROSPERO registration number:**

CRD42021224893.

**Strength and limitations of this study**

- This study will provide the first systematic review and meta-analysis for evaluating the clinical efficacy and safety of removing blood stasis and phlegm in the treatment of patients with epilepsy with cognitive impairment.
- Since most randomized controlled trials on removing blood stasis and phlegm in the treatment of patients with epilepsy with cognitive impairment have small sample size, the present study will provide more reliable evidence for clinical management.
- The reliability of the results will largely depend on the methodological quality of primary studies included and the heterogeneity among them.

## 1 Introduction

Epilepsy is a chronic brain disease that causes the abnormal synchronization of brain neurons and causes the emergence of central neurological dysfunction. About 70 million people worldwide are affected by seizures, and more than a quarter of them can develop intractable epilepsy, while nearly 70 million new people are still in total in the total number of people with epilepsy in China. Over the past 50 years, we have made great progress in understanding epilepsy.^[1]^

Chronic epilepsy causes great pain in patients, which can shorten life or increase the risk of sudden accidental death. In addition, epileptic patients also have prominent neuropsychological, mental and social barriers, such as cognitive impairment, physical disorder, abnormal social behavior, depression, anxiety, etc, which leads to a series of social problems such as limiting employment, reducing marriage rates and reducing quality of life.^[2]^

Cognitive impairment is one of the most common complications of chronic epilepsy, which is associated with complications.^[3]^

Currently, epilepsy therapy is still mainly based on a long-term drug antiepileptic drug (AEDs), which generally claims that no more than 3 AEDs are used in the same way. Because the effects of AEDs on epilepsy patients have been closely observed by the clinical institute, it is also a controlled factor for judging epilepsy with cognitive dysfunction. According to the report, the long-term clinical application of most traditional AEDs can lead to common drug resistance or drug reaction, and even the decline of cognitive memory function in epilepsy patients. Because the pathological mechanism of current epilepsy is not clear, the long-term seizure of chronic epilepsy is difficult to fully control, and the damage caused by the AEDs side reaction to the large population is not possible, and the present situation is unable to meet the current demand for the drug, so it is especially important to find new and effective AEDs that effectively alleviate cognitive impairment.^[4–10]^

Traditional Chinese medicine (TCM) therapy in clinical disease treatment due to positive efficacy, less toxic side effects and other advantages, so the majority of patients and medical workers praised highly accepted.

There are many literatures stating that the efficacy and safety of combined traditional Chinese and western medicine in the treatment of epilepsy with cognitive impairment is better than that of western medicine alone,^[13–22]^ The purpose of this systematic review and meta-analysis is to assess the clinical efficacy and safety of removing blood stasis and phlegm in the treatment of patients with epilepsy with cognitive impairment with a medical basis for inquiry.

## 2 Methods

### 2.1 Inclusion criteria for study selection

#### 2.1.1 Patients and public involvement

Patients and public will not be involved in this study. The protocol follows the Cochrane handbook for preferred reporting items for systematic reviews and meta-analyses protocol statement guidelines. We will describe the changes in our full review if needed. The study will be started on January 1, 2021.

#### 2.1.2 Types of studies

We will include all randomized clinical trials that evaluate the clinical efficacy and safety of removing blood stasis and phlegm in the treatment of patients with epilepsy with cognitive impairment. No restrictions will be imposed on study dates or publication language, type, and status.

However, we will not consider literatures of animal studies, case report, case series, uncontrolled studies, nonclinical trials, non-RCTs, and quasi-RCTs.

#### 2.1.3 Types of participants

Any patients who were diagnosed as epilepsy with cognitive impairment will be included irrespective country, race, age, gender, educational background, economic status and duration, and severity of epilepsy with cognitive impairment.

#### 2.1.4 Types of interventions

In the experimental intervention group, TCM of removing blood stasis and eliminating phlegm combined with other TCM combined with antiepileptic western medicine will be used. However, we will not consider the specific prescription, drug addition and subtraction, dosage form and dose.

The control intervention group was treated with western AEDs alone. However, we will not consider the types of Western medicine, dose, and treatment time.

### 2.2 Types of outcome measures

#### 2.2.1 Major outcomes

1. Clinical efficacy of epilepsy;
2. EEG improvement rate;
3. MOCA score;
4. QOLIE-31 cognitive function score.

#### 2.2.2 Secondary outcomes

1. TCM symptom score;
2. Incidence of adverse reactions;
3. Frequency of seizures of epilepsy;
4. Duration of seizure of epilepsy.

### 2.3 Search methods for the identification of studies

#### 2.3.1 Electronic searches

**Figure 1:**
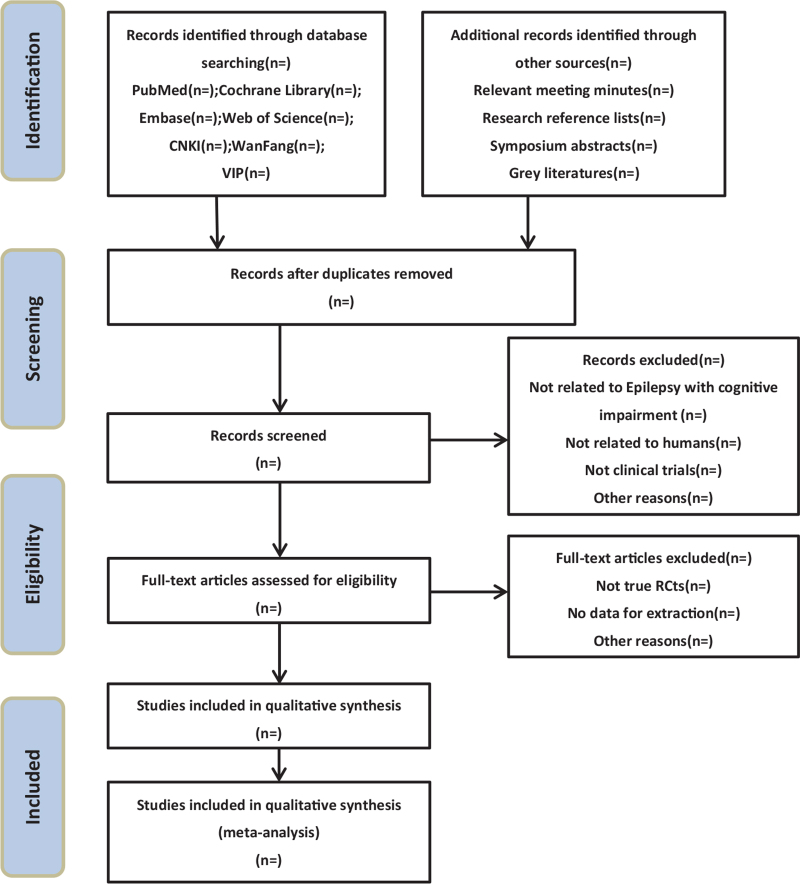
Flow chart of study selection process. Is the flow chart of literature selection process for meta-analysis (http://www.prisma-statement.org).

We will search the Cochrane Library, EMbase, Pubmed, Web of Science, Chinese Journal Full-Text Database (CNKI), Wanfang Database, and VIP database. We will not apply any restrictions to the language and publication date. All randomized controlled trials (RCTs) about the efficacy and safety of removing blood stasis and phlegm in the treatment of epilepsy with cognitive impairment will be included. Search words “stasis, phlegm, phlegn- stasis blocking, epilepsy with cognitive impairment, epilepsy”, the literatures involved are those delivered from the time when the databases were established to January 2021. The search terms in the Chinese database will be the translations of the above words. A preferred reporting items for systematic reviews and meta-analyses flowchart will be created to show the number of articles identified, screened, included, and excluded, reasons for exclusion, and to confirm eligible studies.^[24,25]^ The study selection process will be described in a preferred reporting items for systematic reviews and meta-analyses flowchart (Fig. 1; http://www.prisma-statement.org).

#### 2.3.2 Other resources search

Relevant meeting minutes, eligible research reference lists, symposium abstracts, and gray literature such as degree papers, conference papers will be manually searched for additional resources.

### 2.4 Data acquisition and analysis

#### 2.4.1 Selection of studies

We will import all identified literatures into EndNote X7 software to delete any duplicates. Two authors will screen the titles and abstracts of all potential studies to remove studies that are not related to the topic. Then, full-text of remaining studies will be read carefully to select the final literatures that meet the included criteria. If necessary, a third author will help to solve any divergence between 2 authors.

#### 2.4.2 Data extraction and management

Two researchers will screen all selected articles and extract the data independently. Excel spreadsheet will be used to extract relevant data, including the first author, year of publication, the observation period, the number of participants, the intervention methods of the treatment group and the control group, outcome measures, study results, and adverse events. If there are disagreements, they will settle through discussion. If necessary, the divergence will be discussed with the third author.

#### 2.4.3 Assessment of risk of bias in included studies

By two researchers to quality assessment of included in the document, using the Cochrane risk bias evaluation, including selection bias (the generation of random sequence and allocation concealment), implement bias (blind) of researchers and subjects, measurement bias (research result evaluation method for the blind), follow-up bias (outcome data integrity), reporting bias (selective reports the results of the study), other bias for each index using “low bias”, “not clear”, “highly bias”. If there are different opinions, discuss them. If there is still a disagreement, a third author will be consulted.

#### 2.4.4 Measures of treatment effect

For continuous data, the extracted data will be assessed using a standard mean difference or mean difference of 95% confidence interval. For dichotomous outcomes, we will choose the effect scale indicator relative risk ratio or odds ratio with 95% confidence interval to represent.

#### 2.4.5 Missing data

The researchers will contact the first author by email for further information about the studies while there are missing data. If the missing data is still not obtained in the above way, we will analyze the available data. Furthermore, we will also discuss the potential impact of the missing data.

#### 2.4.6 Assessment of heterogeneity

Statistical heterogeneity across included trials will be examined using chi-squared test and I^2^ test. If I^2^ > 50%, *P* < .1, implies considerable heterogeneity, and a random-effects model will be exploited.

While I^2^ ≤ 50%, *P* > .1, manifests acceptable homogeneity, and a fixed-effects model will be exploited.

#### 2.4.7 Assessment of publication biases

If there are more 8 articles in the meta-analysis, a funnel plot will be established to assess the publication bias. Begg and Egger tests will be used to help assess the symmetry of funnel plot.

#### 2.4.8 Data analysis

Review Manager software version 5.3 and Stata 13.0 statistical software will be used for synthesis and analysis of the data. If there is no heterogeneity (I^2^ ≤ 50%, *P* > .1), a fixed-effects model will be used for analysis. Otherwise (I^2^ > 50%, *P* < .1), a random-effects model will be used for meta-analysis. If there is reasonable heterogeneity, we will conduct a meta-analysis when ample data is extracted from sufficient RCTs. On the other hand, a subgroup analysis will be explored to identify any possible sources of obvious heterogeneity. Under such situation, if it is impossible to perform meta-analysis, we will carry out a narrative synthesis to explain the findings.

#### 2.4.9 Subgroup analysis

If heterogeneity exists in the study results, a subgroup analysis will be conducted to explore the reasons for the existence of heterogeneity from the aspects of participants’ characteristics, different control interventions, outcome measures, etc.

#### 2.4.10 Sensitivity analysis

We will undertake sensitivity analysis to assess the robustness of results by removing high risk of bias studies when significant heterogeneity exists.

#### 2.4.11 Grading the quality of evidence

The grading of recommendations assessment, development, and evaluation standard will be used to evaluate the quality of evidence by two independent authors. The grading of recommendations assessment, development, and evaluation system divides the quality of evidence into four levels: high, moderate, low, and very low. Any disagreements will be solved by a third author through discussion.^[26]^

#### 2.4.12 Ethics and dissemination

There is no necessity for this study to acquire an ethical approval, since no private information of participants will be involved. Results of the present study will be disseminated in a peer-reviewed journal or conference presentation. Important protocol amendments will be documented and updated on PROSPERO.

## 3 Discussion

Epilepsy with cognitive impairment is called “epilepsy disease”^[27,28]^ in TCM. Epilepsy with cognitive impairment is a chronic disease with repeated seizures. After a long period of illness, it enters into collaterals, prevents the flow of qi and blood, and stagnates into blood stasis, making phlegm and blood stasis mutually to become a disease; the viscera is the brain disease; the essential pathogenesis is “meta god out of control”. Asthenia in origin and asthenia in superficiality. The sputum and blood stasis were considered as the pathological products. A large number of literatures also show that the pathogenesis of stagnant blood or phlegm runs through the pathogenesis of epilepsy and cognitive disorders.

Literature has shown that TCM of eliminating phlegm and removing blood stasis have sedative and analgesic effects on nervous system, enhancing memory ability, improving cognitive impairment, antidepression and alleviating ischemia-reperfusion injury. Inhibits seizures, thereby protecting the nervous system. In addition, TCM treatment has fewer side effects, longer duration of action and better curative effect, which opens up new ideas and methods for the treatment of epilepsy combined with cognitive impairment.^[4,5,19]^

Therefore, we attempted to conduct a systematic review and meta-analysis to provide high-quality evidence for the clinical efficacy and safety of removing blood stasis and resolving phlegm in the treatment of epilepsy with cognitive impairment. This study may have some shortcomings. For example, languages other than Chinese and English will be restricted, which will lead to some bias. In addition, the variety of age, dosage, and course of treatment, as well as the number and quality of the literature, may result to significant clinical heterogeneity. We also hope that there will be more high-quality RCTS of TCM for the treatment of epilepsy combined with cognitive impairment in the future.

## Author contributions

**Conceptualization:** YangYang Yu.

**Data curation:** YangYang Yu, Chao Gu.

**Formal analysis:** YangYang Yu, Chao Gu, CanXing Yuan.

**Funding acquisition:** Chao Gu.

**Methodology:** Chao Gu, CanXing Yuan.

**Project administration:** YangYang Yu.

**Resources:** YangYang Yu, Chao Gu.

**Software:** YangYang Yu, Chao Gu.

**Supervision:** CanXing Yuan.

**Writing – original draft:** YangYang Yu.

**Writing – review & editing:** Chao Gu, CanXing Yuan.
